# A Novel Adaptive Sand Cat Swarm Optimization Algorithm for Feature Selection and Global Optimization

**DOI:** 10.3390/biomimetics9110701

**Published:** 2024-11-15

**Authors:** Ruru Liu, Rencheng Fang, Tao Zeng, Hongmei Fei, Quan Qi, Pengxiang Zuo, Liping Xu, Wei Liu

**Affiliations:** 1 College of Information Science and Technology, Shihezi University, Shihezi 832000, China; 2 College of Medicine, Shihezi University, Shihezi 832000, China; 3 College of Science, Shihezi University, Shihezi 832000, China

**Keywords:** feature selection, global optimization, sand cat population optimization, artificial intelligence

## Abstract

Feature selection (FS) constitutes a critical stage within the realms of machine learning and data mining, with the objective of eliminating irrelevant features while guaranteeing model accuracy. Nevertheless, in datasets featuring a multitude of features, choosing the optimal feature poses a significant challenge. This study presents an enhanced Sand Cat Swarm Optimization algorithm (MSCSO) to improve the feature selection process, augmenting the algorithm’s global search capacity and convergence rate via multiple innovative strategies. Specifically, this study devised logistic chaotic mapping and lens imaging reverse learning approaches for population initialization to enhance population diversity; balanced global exploration and local development capabilities through nonlinear parameter processing; and introduced a Weibull flight strategy and triangular parade strategy to optimize individual position updates. Additionally, the Gaussian–Cauchy mutation strategy was employed to improve the algorithm’s ability to overcome local optima. The experimental results demonstrate that MSCSO performs well on 65.2% of the test functions in the CEC2005 benchmark test; on the 15 datasets of UCI, MSCSO achieved the best average fitness in 93.3% of the datasets and achieved the fewest feature selections in 86.7% of the datasets while attaining the best average accuracy across 100% of the datasets, significantly outperforming other comparative algorithms.

## 1. Introduction

During the big data era, with the sharp increase in feature dimensions in intelligent information processing tasks, a large number of unnecessary or unrelated features appear in the dataset, which not only results in reduced model accuracy but also adds to the computational time burden [1]. Feature selection (FS), as a key data dimensionality reduction method, aims to select the most informative feature subset in a given feature set to improve the performance of machine learning models [2]. FS helps to cut down on computational costs while also increasing the model’s interpretability. Especially when dealing with large-scale datasets, it can significantly improve computational efficiency and reduce the complexity of model training.

The traditional feature selection methods can be divided into the filtering method, wrapping method, and embedding method [3]. Filtering methods are selected by independent evaluation of each feature, with common evaluation metrics including information gain (IG), the chi-square test, and mutual information (MI). These methods are computationally efficient but often ignore the correlation between features. By combining feature subsets with specific learning algorithms to evaluate their performance, the package method selects the optimal feature subsets. Although the wrapping method can consider the relationship between features, it is computationally expensive, especially when the feature dimension is high. The embedding method integrates the feature selection process into the model training process and directly selects features through regularization technology or model structures, such as ridge regression (RR) and decision trees (DTs), etc. This method has a good performance in computational efficiency and accuracy.

When it comes to handling large-scale datasets or high-dimensional data, the drawbacks of these traditional methods become particularly prominent. Hence, researchers have embarked on seeking more flexible algorithms capable of handling complex feature interactions. Metaheuristic algorithms, as an emergent approach, have emerged as an effective tool for resolving feature selection issues with their potent global search capabilities and adaptability to complex matters [4].

Metaheuristic algorithms draw inspiration from natural processes and human behavioral patterns. Based on the heuristic approach mechanism, these algorithms fall into five categories, namely evolutionary-based algorithms, population-based algorithms, physics/chemistry-based algorithms, human behavior-based algorithms, and mathematics-based algorithms. Evolutionary-based algorithms mimic natural selection and genetic processes, with typical instances including Genetic Algorithms (GAs) [5], Differential Evolution (DE) [6], and Evolutionary Strategies (ESs) [7]. Population-based algorithms simulate the behavior of biological populations, such as the Krill Herd Algorithm (KHA) [8], the Honeybee Algorithm (HBA) [9], and the Fox Optimization Algorithm (FOX) [10]. Physics/chemistry-based algorithms draw from physical and chemical phenomena in nature, like Simulated Annealing (SA) [11], the Crystal Structure Algorithm (CryStAl) [12], and the Gravitational Search Algorithm (GSA) [13]. Human behavior-based algorithms borrow characteristics of human thinking, behavior, or social structures, such as the Love Evolutionary Algorithm (LEA) [14], the Rider Optimization Algorithm (ROA) [15], and the Stadium Audience Optimization Algorithm (SSO) [16]. Finally, mathematics-based algorithms are designed based on mathematical theories, such as Hyperbolic Sine Cosine Optimization (SCHO) [17], Triangular Topology Aggregation Optimization (TTAO) [18], and Exponential Distribution Optimization (EDO) [19].

According to the “No Free Lunch Theorem” for optimization [20], no single algorithm performs best in every situation, which explains the continuous emergence of diverse optimization algorithms.

The Sand Cat Swarm Optimization (SCSO) algorithm was proposed in [21], which mimics how sand cats survive in the natural world, characterized by its simple principle, few parameters, and strong global search capability. This algorithm has shown excellent performance on 20 famous test functions and 10 CEC2019 test functions and has performed well in seven engineering design problems. However, the original algorithm still encounters issues such as limited optimization accuracy, slow convergence, and a tendency to become trapped in local optima when addressing high-dimensional highly intricate optimization tasks. To overcome these issues, several improvements have been made to SCSO, and the enhanced version is named MSCSO.

This study makes the following key contributions:

1. An innovative multi-objective feature selection method, the improved Sand Cat Swarm Optimization algorithm, is proposed for the feature selection problem. This method enhances feature selection efficiency while considering both the classification error rate and feature selection ratio as objectives, aiming to obtain the optimal feature subset, reduce redundant features, and improve classification accuracy.
2. The population initialization method, nonlinear parameter handling, and position update strategy have been innovatively improved, and the Gaussian–Cauchy mutation strategy has been introduced. These enhancements significantly boost the algorithm’s global search capability, convergence speed, and optimization accuracy, while effectively avoiding the issue of local optima.
3. The global optimization performance of MSCSO was evaluated through the CEC2005 benchmark test, with results showing that it performed excellently on 65.2% of the test functions. Additionally, in feature selection experiments conducted on 15 datasets from UCI, MSCSO achieved the optimal average fitness in 93.3% of the datasets, demonstrated the optimal number of feature selections in 86.7% of the datasets, and reached the highest average accuracy in 100% of the datasets, all superior to other comparative algorithms.

The structure of the remainder of this study is as follows: Section 2 examines the relevant research progress in feature selection, Section 3 describes the improvement methods of SCSO and its complexity analysis, Section 4 presents the analysis and discussion of experimental results, and Section 5 reviews the key findings of this research and suggests potential avenues for future investigation.

## 2. Related Work

### 2.1. Feature Selection Based on Traditional Methods

Feature selection (FS) is regarded as an NP-hard problem because of the complexity of its search space. With increasing data dimensionality, the number of potential feature subsets increases exponentially, making it extremely challenging to identify the optimal subset. To tackle this issue, researchers have developed numerous approaches to feature selection, which are typically divided into three main types, namely filtering methods, wrapper methods, and embedded methods.

Filter, wrapper, and embedded methods are three widely used feature selection techniques, each with distinct characteristics. Filter methods evaluate the relationship between features and the target variable to screen feature subsets, operating independently of model training. Univariate methods are computationally efficient but may overlook interactions among features, while multivariate approaches can capture complex inter-feature relationships. For example, Ref. [22] utilized variance–covariance distance to eliminate features with minimal variance for dimensionality reduction, while Ref. [23] employed MI and the variance inflation factor (VIF) to eliminate multicollinearity, thereby refining the feature set. Wrapper methods, on the other hand, evaluate feature subsets based directly on model performance, using metrics such as forward selection, backward elimination, and recursive feature elimination (RFE). Ref. [24] applied forward feature selection to iteratively construct transitional index maps, and Ref. [25] combined recursive feature elimination with cross-validation (RFECV) to optimize feature selection for decision tree models. Embedded methods perform feature selection during model training, allowing for synchronous feature selection and model fitting. For instance, Ref. [26] leveraged LASSO to select features highly correlated with liver disease, enhancing classification accuracy. Additionally, Ref. [27] employed multivariate variational mode decomposition (MVMD) and kernel extreme learning machine (KELM) models to predict plant and soil moisture evaporation, effectively addressing complex feature selection tasks.

### 2.2. Feature Selection Based on Metaheuristic Algorithm

In traditional feature selection methods, the filtering method, wrapper method, and embedded method each have their strengths and weaknesses. Although these methods perform well in addressing feature selection issues, they may still encounter limitations when dealing with high-dimensional data, complex interactions between features, and limited computational resources. To overcome these limitations, researchers are gradually turning to metaheuristic algorithms. These algorithms simulate heuristic processes in the natural world and can effectively search the feature space to find feature subsets suitable for specific problems.

The introduction of meta-heuristic algorithms provides a new way to solve the difficult problem of feature selection, especially in the case of high feature dimensionality or complex relationships between features. Next, the application of several typical meta-heuristic algorithms in feature selection will be discussed. Ref. [28] proposed the binary arithmetic optimization algorithm (BAOA) to address the feature selection problem. The algorithm transforms continuous variables into discrete variables through S-shaped and V-shaped shift functions and combines four logical operations and parametric models based on sine and cosine functions to optimize the feature selection process. Ref. [29] adopted an improved slime mold algorithm (ISMA) for feature selection. This algorithm significantly improves classification performance by simultaneously optimizing parameters and feature selection of a support vector machine (SVM). Ref. [30] used the Binary Macaque Optimization Algorithm (BMOA) for feature selection, which improves classification accuracy by screening for the most relevant and effective features. The improved version of the BMOA (IBMOA) further enhances exploration and development capabilities. Ref. [31] designed an enhanced raccoon optimization algorithm (mCoatiOA) to enhance its exploration and development capabilities by introducing adaptive s-best mutation operators, directional mutation rules, and global optimal search control. Ref. [32] improved the Gray Wolf Optimization algorithm (GWO) for feature selection, improved the initial population quality by introducing the ReliefF algorithm and Coupla entropy, and optimized the search process using competition guidance and DE’s lead wolf enhancement strategy. This method effectively reduces the feature set and enhances classification accuracy. Ref. [33] presented an advanced variant of the Harris Eagle Optimization algorithm (EHHO). By introducing a hierarchical structure, the algorithm shows a stronger capacity for handling complex problems. In feature selection, EHHO enhances classification performance, decreases feature count, and cuts down on execution time. The above algorithms perform well in feature selection, but there are some limitations in practical applications. First, they often require selecting a large number of features, which results in low computational efficiency, especially when dealing with large-scale datasets, leading to slower processing speeds. Additionally, due to their high computational cost and sensitivity to parameters, these algorithms have limited practicality and are difficult to apply in real-time systems or complex environments.

Some scholars use hybrid algorithms for feature selection, which combine two or more different algorithms to take full advantage of each. Ref. [34] proposed the mixed sine–cosine Firehawk algorithm (HSCFHA). The model combines the Fire Eagle algorithm (FHO) and the sine and cosine algorithm (SCA) to obtain better results in feature selection problems by exploiting the advantages of both algorithms in exploration and development. Ref. [35] used a particle swarm-guided Condor search algorithm (PS-BES) for feature selection. The algorithm guides the condor’s search by combining the speed of the particle swarm and utilizes the attack–retreat–surrender technique to increase the capacity for escaping local optima, thus improving the overall effect of the feature selection process. Ref. [36] created a new integrated binary metaheuristic that combines the Deep Throat Optimization algorithm (DTO) and the SCA. By using the SCA to improve the exploration process and combining DTO to accelerate convergence, the algorithm improves the efficiency and accuracy of feature selection. Ref. [37] combined particle swarm optimization (PSO) and the firefly algorithm (FA), combining the advantages of each algorithm for problems related to feature selection. Ref. [38] proposed a hybrid optimization approach (GWDTO) based on GWO and DTO for feature selection. This method improves the effectiveness of feature selection by effectively balancing the exploration and development steps in the optimization process. These hybrid algorithms perform well in feature selection, but there are some issues. Firstly, they have high computational complexity, leading to low efficiency when processing large datasets. Secondly, they are prone to getting trapped in local optima, making it difficult to find the global optimum. Finally, the algorithms are sensitive to parameters and tuning them is challenging, which makes them less flexible and practical in real-world applications.

Compared to other complex meta-heuristic algorithms, SCSO has the advantage of fewer parameters, making it easier to adjust. This results in a simpler optimization process and reduced complexity in parameter tuning. Additionally, SCSO maintains a good balance between exploration and exploitation, effectively avoiding local optima. By simplifying the adjustment process, SCSO improves feature selection efficiency and adapts well to complex datasets. Therefore, SCSO was chosen as the base algorithm for improvement in this study.

### 2.3. Motivation

Feature selection is a critical step in machine learning and data mining, aimed at removing irrelevant features to ensure model accuracy, thereby improving interpretability and generalization. However, existing feature selection methods have notable limitations, necessitating more efficient and accurate solutions.

(1) Traditional feature selection methods struggle with feature correlation and computational efficiency. Filter methods, though simple, overlook correlations between features; wrapper methods consider feature relationships but are computationally intensive, making them unsuitable for high-dimensional data; and embedded methods are often affected by model dependency. Addressing these issues requires an efficient method capable of accurately identifying feature correlations.
(2) In heuristic algorithm applications, many algorithms exhibit low accuracy and fail to identify the optimal subset in feature selection, limiting their practical utility. Thus, designing superior optimization strategies to improve accuracy and applicability has become a current research focus.
(3) Additionally, heuristic algorithms in feature selection often suffer from premature convergence, getting trapped in local optima and failing to reach the global optimum, thereby limiting their effectiveness. The key to overcoming this lies in enhancing convergence speed and solution diversity to ensure a comprehensive search and optimal solutions.

To address these issues, this study proposes a feature selection technique based on MSCSO. This algorithm minimizes redundant features while balancing error rates and the ratio between selected and original features, aiming for both accuracy and efficiency in feature selection. The proposed improvements include diversified population initialization via logistic chaotic mapping and lens imaging reverse learning, nonlinear parameter adjustment, and individual position updates optimized with Weibull flight and triangular marching strategies. Additionally, Gaussian–Cauchy mutation is integrated to enhance the algorithm’s ability to escape local optima. These enhancements provide a robust mechanism for feature selection, promising to improve stability and effectiveness in practical applications.

## 3. Improved Sand Cat Swarm Optimization Algorithm

### 3.1. Improved Population Initialization

As one of the most classical chaotic mapping methods, logistic chaotic mapping enhances the diversity of initial solutions, helping the algorithm perform a more effective global search. The formula for logistic chaotic mapping is given by

(1) $$X_{t+1}=\mu X_{t}(1-X_{t})$$

where $\mu \in \left(0,4\right]$, t represents the current iteration count, and $X_{t}\in \left(0,1\right)$.

Reverse learning improves the search capability of the algorithm by generating the reverse solution of the current solution and enlarges the search space of the algorithm. Lens imaging reverse learning is an improvement of reverse learning. By simulating the imaging principle of optical lenses, the reverse solution of the solution is generated so as to further enhance the variability within the population and speed up the algorithm’s convergence. The formula is shown as follows:

(2) $$X’=\frac{ub+lb}{2}+\frac{ub+lb}{2k}-\frac{X}{k}$$

where ub denotes the solution’s upper bound and lb its lower bound. The term $\frac{ub+lb}{2}$ in the formula represents the center of the solution space, which is the average of the upper and lower bounds. The terms $\frac{ub+lb}{2k}$ and $\frac{X}{k}$ adjust the current solution X through the scaling factor k. In this study, k is set to 0.75. When k=1, the formula reduces to standard opposition-based learning; when k≠1, by simulating the principle of optical lens imaging, the formula changes the distribution range of the opposite solutions, further enhancing the diversity of the population.

First, the initial sand cat population was generated by logistic chaos mapping, and then the corresponding reverse population was generated by lens imaging reverse learning. The two populations were combined, the evaluation of each individual’s fitness value was performed, and they were ranked from smallest to largest. Next, the top N individuals with the highest fitness scores were chosen to constitute the final sand cat population. Compared with the original SCSO, this approach not only accelerates the search process but also greatly increases the search space, thereby enhancing the algorithm’s capability to perform a global search.

### 3.2. Nonlinear Parameterization

The foraging behavior of sand cats consists of two main stages, namely search and attack. During the search phase, the sand cat conducts global exploration, scanning the entire feasible domain for potential targets. In the attack phase, fine-grained search is focused on a specific area, similar to local optimization. How to coordinate the switching between these two stages effectively is the key to improve the effectiveness of the algorithm. This coordination process relies on the control of the key parameter R. The parameter R is directly related to rg. In the original SCSO algorithm, rg decreases linearly from 2 to 0 as the number of iterations increases. However, to enhance the method’s global exploration capability and avoid local optima traps, the parameters need to be nonlinearized, increasing the number of iterations during the search stage. This helps prevent premature convergence and improves the algorithm’s ability to escape local optima. The specific formula is as follows:

(3) $$rg=\left(\frac{S}{\log_{10}^{S}}\right)\times \log_{10}^{{\left(2-\frac{t}{T_{\max}}\right)}^{2}}$$

where the initial value of S is 2, t represents the current iteration number, while $T_{\max}$ denotes the maximum iterations. The change in r is associated with rg, r is the sensitivity range of each sand cat, rand is a value randomly chosen between 0 and 1, and r is calculated by the following formula:

(4) r=rg×rand

### 3.3. Enhanced Location Update Strategy

When $\left|R\right|$ > 1, the sand cat begins searching for prey. The sand cat adjusts its position according to a random candidate location $P_{bc}(t)$, the current location $P_{c}(t)$, the range of sensitivity r, and a rand value between 0 and 1. The location update formula is given by

(5) $$P(t+1)=r(P_{bc}(t)-rand\cdot P_{c}(t))$$

During the prey search phase, the location is updated mainly by the random candidate location $P_{bc}(t)$ and current location $P_{c}(t)$. If the individuals in the algorithm are too concentrated in the position that is currently considered to be optimal, especially if this position is only a local optimal solution, the amplitude of the overall disturbance will gradually decrease as the iteration progresses. This will cause the whole population to become more and more close to this local optimal region, which will significantly reduce the variety within the population, causing the algorithm to be prone to premature convergence and eventually affecting the optimization accuracy. To solve this problem, the Weibull flight strategy is designed to create new individual locations to introduce more variability into the search process and prevent getting trapped in a local optimal solution prematurely. In searching for prey, the position update formula combined with Weibull’s flight strategy is as follows:

(6) $$P_{new}(t+1)=r\cdot R_{w}\cdot (P_{bc}(t)-rand\cdot P_{c}(t))$$

where $P_{bc}(t)$ is the random candidate location, $P_{c}(t)$ is the current location, r is the sensitivity range, and $R_{w}$ is a random value drawn from a Weibull distribution. The probability density formula for the Weibull distribution is given by

(7) $$f(x,\lambda ,k)=\left(\frac{k}{\lambda }\right)\times {\left(\frac{x}{\lambda }\right)}^{k-1}\times \exp\left(-{\left(\frac{x}{\lambda }\right)}^{k}\right)$$

where x is the value of the random variable, λ is the scale parameter, k is the shape parameter, and the values of λ and k determine the shape and scale of the distribution. In this study, λ is 0.5 and k is 4.

When $\left|R\right|\leq 1$, the sand cat attacks its prey by first using the best location $P_{b}(t)$ and the current location $P_{c}(t)$ to generate a random location $P_{rnd}$. Assuming that the sensitivity range of the sand cat is a circle, the roulette method is used to randomly select an angle θ for each sand cat, where the range of θ is from 0 degrees to 360 degrees, and finally, the prey is attacked by Equation (9). To maintain proximity to the prey, the algorithm uses random positioning for the sand cat. Additionally, random angles are used to avoid local optima entrapment.

(8) $$P_{rnd}=\left|rand\cdot P_{b}(t)-P_{c}(t)\right|$$

(9) $$P(t+1)=P_{b}(t)-r\cdot P_{rnd}\cdot \cos(\theta )$$

In attacking prey, the continuous local search can lead to a decrease in population diversity, thus making the algorithm lose enough exploration ability to discover new solution space regions. To address this issue, the triangle parade strategy was added. By introducing a random walk element into local search, the triangular parade enhances the exploration of the algorithm, making it more likely to escape local optima, and helps the sand cat to explore in a wider solution space. The formula for updating positions combined with the triangular parade strategy is as follows:

(10) $$P_{new}(t+1)=P_{b}(t)+r\cdot alph$$

where $P_{b}(t)$ is the best position, r is the sensitivity range, and alph is the offset of the new position. The formula for alph is

(11) $$alph=L^{2}+LP^{2}-2\times LP\times L\cdot \cos(2\times \pi \times rand)$$

(12) LP=L×rand

(13) $$L=P_{b}(t)-P_{c}(t)$$

where L represents the distance from the sand cat to its current best solution, LP is used to introduce randomness, and rand is a random number between 0 and 1.

### 3.4. Out of Optimal Value

After one iteration, Gaussian and Cauchy variations were used to disturb the optimal position of the sand cat population and update the position of the whole population. Gaussian variation helps individuals fine-tune their position in the solution space by introducing small amplitude random changes and enhances local search ability and optimizes solutions more effectively while avoiding premature convergence. In contrast, Cauchy variation introduces large random perturbations that allow individuals to jump out of local optimal solutions, thereby improving global exploration capabilities, and the formula for the perturbation position of Gaussian–Cauchy variation is

(14) $$P_{h}(t)=P_{b}(t)\times (1+\Delta P(t))$$

(15) ΔP(t)=α×Gaussian(1,0)+β×cauchy(1,0)

(16) $$\alpha =t/T_{\max}$$

(17) β=1−α

where $P_{h}(t)$ is the position after perturbation by Cauchy–Gaussian variation, $P_{b}(t)$ is the best position, t represents the current iteration, $T_{\max}$ denotes the total number of iterations, and Gaussian(1,0) is a random variable based on Gaussian distribution. cauchy(1,0) denotes a random variable that follows a Cauchy distribution.

In order to further optimize the population after the position disturbance of Gaussian–Cauchy variation, an optimal strategy, the greedy strategy, was adopted. By comparing the fitness scores of the modified individual and the original individual, the strategy selects the individual with better fitness. If the mutated individual has better fitness, it replaces the original individual, thereby improving the overall performance of the algorithm and ensuring that each generation moves toward the optimal solution by accurately selecting better solutions and robustly converging toward the global optimum. The formula for the greedy strategy is

(18) $$P_{c}(t)=\left\{\begin{array}{l} P_{c}(t)f(P_{h}(t))\geq f(P_{c}(t))\\ P_{h}(t)f(P_{h}(t))<f(P_{c}(t)) \end{array}\right.$$

where $P_{c}(t)$ is the current position, $P_{h}(t)$ is the position after Cauchy–Gaussian variation perturbation, and f is the fitness function.

### 3.5. MSCSO for Feature Selection

Suppose S is a dataset with K samples and M features and F is a set of M features. The purpose of feature selection is to optimize the objective function by selecting the optimal feature subset $Y_{b}(Y_{b}\in M)$, since the setting of the objective function is to maintain a balance between minimizing the error rate and minimizing the number of features selected. Therefore, this study chooses the linear combination of error rate and the number of selected features as the objective function, and the formula is as follows:

(19) $$f=\lambda \times error+(1-\lambda )\times (1-\frac{Y}{M})$$

In order to apply MSCSO to feature selection, it is necessary to binarize the sand cat individual. The details are shown in Formula (23).

(20) $$Y_{i}=\left(y_{i}^{1},y_{i}^{2},....y_{i}^{M}\right),y_{i}^{j}\in \left\{0,1\right\}$$

where $y_{i}^{j}$ = 1 means that the *j* feature in the i feature subset $Y_{i}$ is selected, and $y_{i}^{j}$ = 0 means that this feature is not selected. The feature selection problem in this study can be expressed as an optimization problem, and the formula is as follows:

(21) $$\left\{\begin{array}{l} \min f(Y)\\ s.t.Y_{i}=(y_{i}^{1},y_{i}^{2},....y_{i}^{M}),y_{i}^{j}\in \{0,1\}\\ j=1,2,....M\\ 1\leq |Y|\leq M \end{array}\right.$$

where minf(Y) is the minimization objective function and s.t. is the constraint.

MSCSO was originally used to solve the problem of continuous variables, but the value of the feature subset is limited to {0, 1}, so to convert continuous values between [0, 1] to binary values, 0.5 is used as the threshold to decide whether to select a feature. When the value in the individual is greater than 0.5, it is converted to 1, indicating that the feature has been selected. When the value is less than or equal to 0.5, it is converted to 0, indicating that the feature is not selected [2,3,31].

### 3.6. Complexity Analysis of MSCSO

The time complexity of this algorithm consists of the initialization phase and multiple steps in each iteration. In the initialization phase, the algorithm first generates 2×N positions and initializes the population using chaotic mapping and opposition-based learning, with a time complexity of O(2×N×D), where N is the number of search agents and D is the feature dimension. Then, it calculates the fitness of each individual and performs sorting, with a time complexity of O(2×N×D+2×Nlog(2×N)). In each iteration, position updates, binarization, fitness calculations, and boundary checks are performed for each individual, with a time complexity of O(N×D). The position update includes calculating random factors, Lévy flight, Weibull flight, and other O(D) operations. Gaussian and Cauchy mutations are applied to all individuals for perturbation, with a complexity of O(N×D). Combined with fitness recalculations and greedy selection, the overall complexity remains O(N×D). Assuming a total number of iterations $T_{\max}$, the overall time complexity is $O(T_{max}\times N\times D)$, with the number of iterations being the primary computational cost.

**Figure 1:**
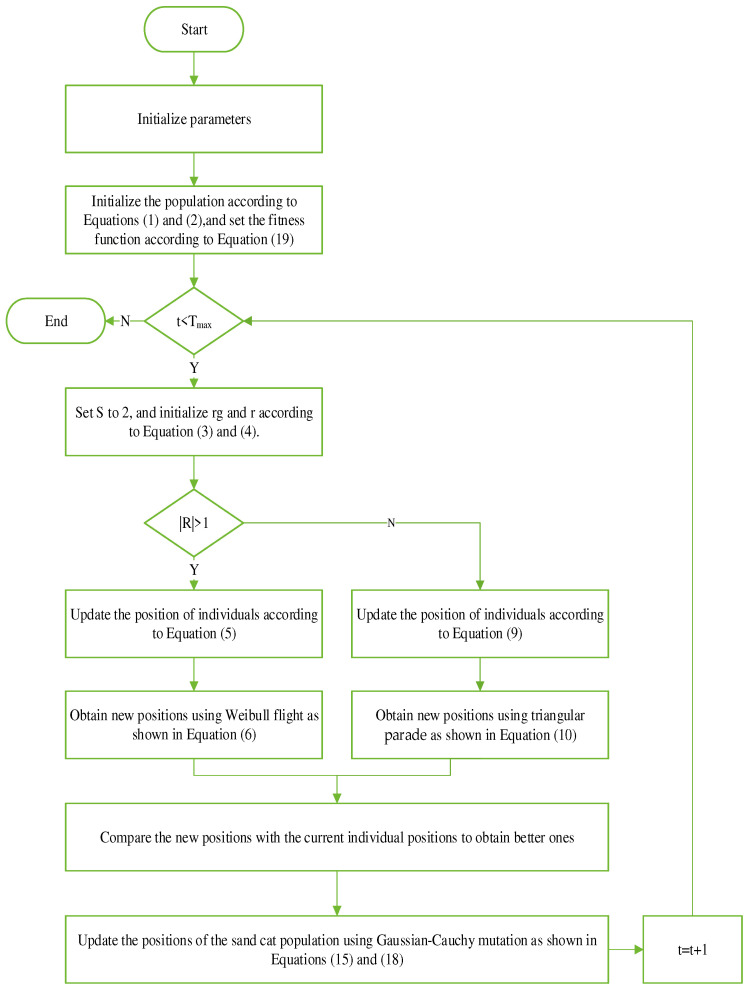
Flow chart of MSCSO optimization algorithm.

In this section, the original sand cat algorithm is mainly improved, and the mathematical model of MSCSO is depicted in Algorithm 1, with the overall flowchart depicted in Figure 1.
**Algorithm 1** Pseudocode for the MSCSO optimization algorithm1: Initialization parameters: population size N, maximum iteration count $T_{\max}$, upper and lower limits of solutions ub and lb. 2: Initialize the optimal position of the sand cat $P_{b}$ and the optimal fitness value $P_{best}$. 3: Fitness function f(x): see Equation (19). 4: Population initialization: to initialize the sand cat population, use Equations (1) and (2) to compute fitness value $f(P_{i})$ of every sand cat individual, and take the first N to form a new sand cat population. 5: While $t<T_{\max}$ do 6: for every individual sand cat $P_{i}$ do 7:  To ensure that each individual sand cat $P_{i}$ is in the limits of the solution and to calculate the $P_{i}$, the fitness value of the $f(P_{i})$ is used. 8:   if $f(P_{i})<P_{best}$, then 9:    Assign the value of $f(P_{i})$ to $P_{best}$, updating the optimal location $P_{b}$. 10:  end if 11: end for 12: Set S to 2 and initialize rg and r according to Equations (3) and (4). 13:  if |R| > 1 then 14:  Update individual positions according to Equation (5). 15:  Use the Weibull flight strategy to obtain a new position, see Equation (6). 16: else 17:  Adjust positions based on Formula (9). 18:  Use the triangle parade to obtain a new position, see Equation (10). 19: end if 20: Calculate the fitness of the individual value $f(P_{i})$ and the new position of fitness value $f(P_{newi})$. 21: if $f(P_{newi})<f(P_{i})$, then 22:   Assign $f(P_{newi})$ to $P_{i}$. 23: end if 24: Use Formulas (15) and (18) to update the location of the entire sand cat population. 25: Use Formula (19) to ensure excellence. 26: If the number of iterations increases by 1, t = t + 1. 27: end while

## 4. Performance Metrics of Experiment

Two key experiments are conducted in this section to thoroughly examine the effectiveness and precision of the MSCSO approach introduced in this research. The first experiment aimed to assess how well the MSCSO algorithm performs on global optimization problems, using the CEC2005 reference function set, which covers a variety of optimization problems from simple to complex, including unimodal functions, multimodal functions, and fixed-dimensional multimodal functions. Through these benchmark functions, we can methodically assess how well the MSCSO algorithm performs and maintains stability across various optimization challenges. The second experiment focused on feature selection, using 15 classical datasets covering different domains and feature selection difficulties. By applying the MSCSO algorithm to these datasets, its performance in actual data processing can be evaluated. Feature selection, as a key step in machine learning and data mining, has a major effect on the performance and complexity of the model, so it is of great significance to evaluate the effect of the MSCSO algorithm in this task. All experiments were conducted on the 64-bit version of MATLAB R2022a to ensure a consistent experimental environment. In these two experiments, the results of the MSCSO algorithm are evaluated against the original SCSO algorithm and several other commonly used meta-heuristic algorithms in detail, and a variety of performance indicators are used to evaluate these algorithms so as to fully understand the relative advantages and application potential of the MSCSO algorithm.

### 4.1. Performance Metrics of Experiment

To assess the effectiveness of MSCSO, the following evaluation indicators were used in this study:

Average fitness: Fitness is a joint evaluation of the classification error rate and the proportion of selected features relative to the total number of features. The smaller the fitness, the better the effect, which means reducing the classification error rate while minimizing the selection of feature count to achieve the optimal effect. The average fitness is calculated as the mean of several separate runs, and its expression is shown as follows:

(22) $$f_{mean}=\frac{\sum_{i=1}^{R}f_{i}}{R}$$

where R is the number of independent runs. In global optimization, R is assigned a value of 30 for the number of independent trials, whereas in feature selection, R is assigned a value of 20 and $f_{i}$ denotes the fitness value obtained from the initial run i.

Standard deviation: This is used to assess the consistency and robustness of the optimization algorithm across multiple runs. A reduced standard deviation indicates a more stable optimization algorithm. The formula is as follows:

(23) $$std=\sqrt{\frac{\sum_{i=1}^{R}{\left(x_{i}-x_{mean}\right)}^{2}}{R}}$$

where R is the number of independent runs and $x_{i}$ and $x_{mean}$ represent the measured value and average value in the i run, respectively.

Average accuracy: this is the average of classification accuracy after the R run, defined as

(24) $$acc=\frac{\sum_{i=1}^{R}acc_{i}}{R}$$

where $acc_{i}$ is the accuracy of the first run of i.

Average feature selection: this represents the mean number of selected features across R runs, defined as

(25) $$feature=\frac{\sum_{i=1}^{R}size(i)}{R}$$

where size(i) indicates the count of features chosen during the i-th run.

### 4.2. Parameter Setting of Algorithm

Table 1 presents the parameter configurations for MSCSO, SCSO, Moth Flame Optimization (MFO) [39], the Salp Swarm Algorithm (SSA) [40], Four-Vector Optimization (FVIM) [41], the Arithmetic Optimization Algorithm (AOA) [42], the SCA [43], PSO [44], Dandelion Optimization (DO) [45], and Sailfish Optimization (SFO) [46]. These settings significantly impact algorithm efficiency and the outcomes of the experiments. Specifically, the table includes the number of iterations, population size, and the specific parameter configurations for each algorithm.

### 4.3. Experiment 1: Global Optimization of CEC2005 Test Functions

This section assesses how well the MSCSO algorithm performs with the CEC2005 benchmark functions, as shown in Supplementary Materials Table S1. Among the 23 test functions, F1 to F7 are unimodal benchmark functions, F8 to F13 are multimodal benchmark functions, and F14 to F23 are fixed-dimension multimodal benchmark functions. Unimodal benchmark functions mainly test the algorithm’s ability to perform local searches, while multimodal benchmark functions examine the algorithm’s ability to escape local optima and find the global optimum. Fixed-dimension multimodal benchmark functions evaluate how well the algorithm performs in a global search at specific dimensions. To thoroughly assess the effectiveness of the MSCSO algorithm, nine commonly used heuristic algorithms are chosen for comparison, including standard SCSO, MFO, the SSA, FVIM, the AOA, the SCA, PSO, DO, and SFO. All algorithms are assessed under the same experimental conditions to maintain the integrity and consistency of the results. Each algorithm uses 30 search agents and performs 1000 iterations. To minimize the effects of random initialization on the results, each algorithm is executed 30 times separately, with the performance assessed by averaging fitness values and calculating the standard deviation.

#### 4.3.1. Numerical and Statistical Analysis of Experiment 1

Table 2 presents a comparison of the mean fitness and standard deviation between MSCSO and existing optimization algorithms for the CEC2005 test functions. It can be seen from the ranking of average fitness that MSCSO outperforms other optimization algorithms on 15 test functions. Specifically, MSCSO performs best on F1–F5, F8–F11, F15, F16, and F20–F23; performs second best on F7, F12, F13, and F14; ranks third for F17, F18, and F19; and has relatively weaker competitiveness in F6. Overall, MSCSO demonstrates the best performance among all compared algorithms.

#### 4.3.2. Comparative Analysis of MSCSO and Other Algorithms on Test Functions

In the CEC2005 test functions, MSCSO performed significantly better than SCSO. Specifically, MSCSO outperformed SCSO in 17 test functions, while SCSO only outperformed MSCSO in F17 and F18, both of which performed equally on F9–F11 and F16. MSCSO vs. MFO: MSCSO performed well on 19 test functions, with the same performance on F16, only underperforming compared to MFO on F17–F19. MSCSO compared to the SSA: the SSA had better average fitness on F6, F14, F17, F18, and F19, with the same average fitness on F16, and MSCSO performed best on the remaining 17 test functions. MSCSO vs. FVIM: MSCSO outperforms FVIM on 23 test functions. MSCSO vs. the AOA: the AOA won only on F7, achieving the same score on F2, F9, and F10, and MSCSO achieved better results on the remaining 19 test functions. MSCSO vs. the SCA: MSCSO is 100 percent better than the SCA and has a lower mean fitness. Comparison between MSCSO and PSO: Among the 23 test functions, MSCSO achieved optimal results in 17 test functions; PSO was superior to MSCSO in F6, F14, F17, F18, and F19, and the two algorithms had the same results in F16. MSCSO vs. DO: DO outperforms MSCSO in F6, F12, F13, F14, F17, F18, and F19 and performs the same in F16, and MSCSO performs better in the remaining 15 test functions. MSCSO vs. SFO: SFO failed to surpass MSCSO in any of the test functions, only scoring the same as MSCSO in F8 and F11.

Table 2 further shows that MSCSO ranks highest among all comparison algorithms, while the SCA ranks lowest. The order is MSCSO > SCSO = DO > FVIM > SSA > PSO > SFO > AOA > MFO > SCA.

Wilcoxon tests are widely used in statistical analysis to compare the differences between two algorithms. Table 3 records the *p*-values of the Wilcoxon test between MSCSO and existing optimization algorithms. These *p*-values indicate whether significant differences exist between the performance of the two algorithms across various test functions. When the *p*-value falls below 0.05, it indicates that the difference between MSCSO and comparison algorithms in this test function is statistically significant. Conversely, if the *p*-value is 0.05 or higher, it suggests that there is no significant performance disparity between the two algorithms for the given test function. *p*-values above 0.05 are shown as underlined below.

#### 4.3.3. Graphic Analysis of Experiment 1

**Figure 2:**
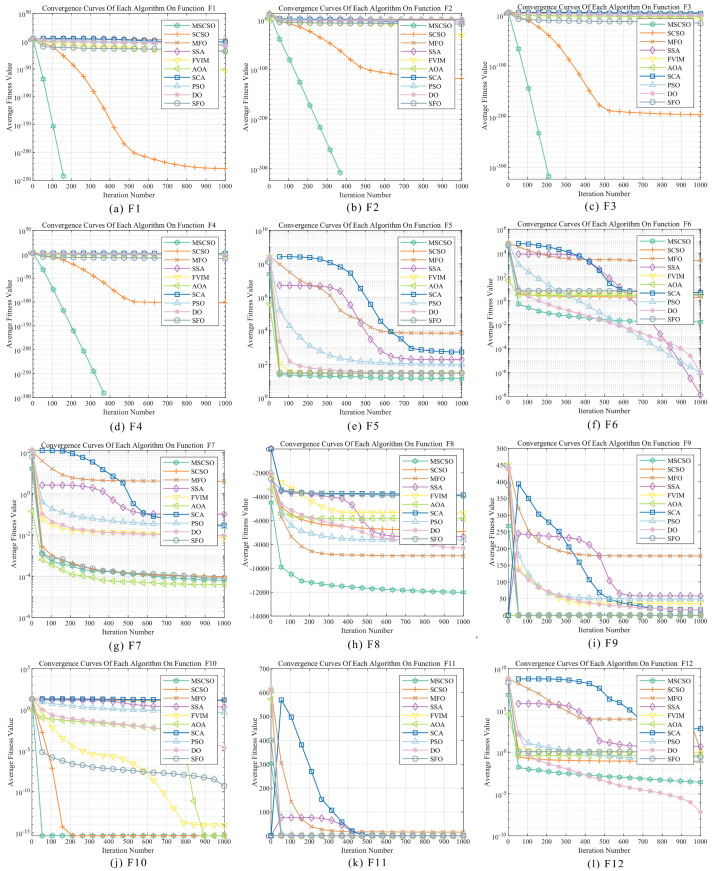

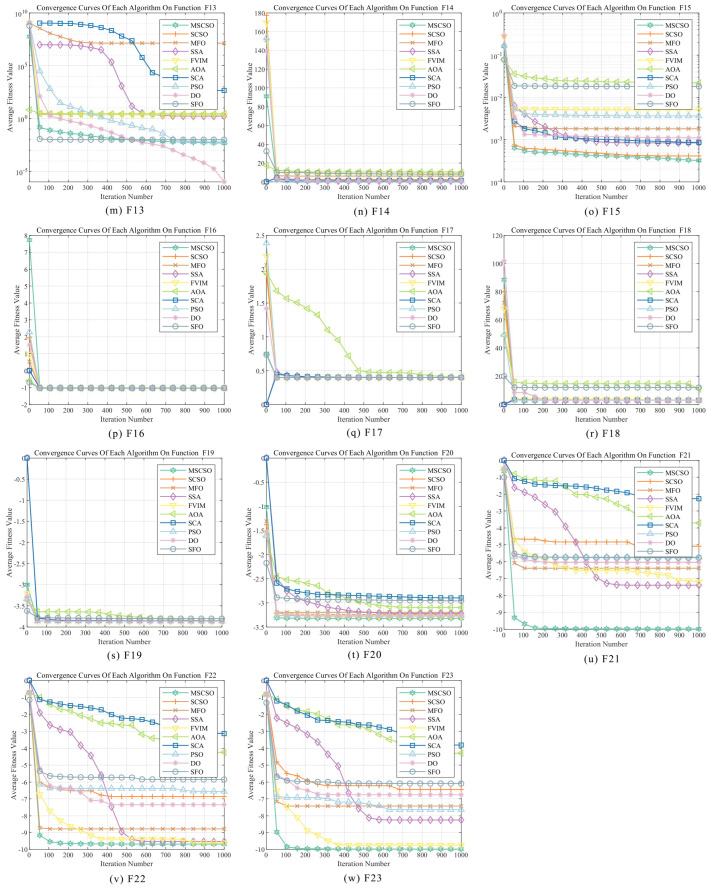
Comparison of convergence curves of MSCSO and other optimization algorithms for global optimization.

This section primarily conducts an in-depth analysis of the performance of various optimization algorithms through convergence curve diagrams, assessing their performance in solving the CEC2005 test functions. By observing the convergence curves, one can intuitively understand the convergence speed and final convergence state of each algorithm during the iteration process. Figure 2 illustrates the convergence behavior of MSCSO compared to other optimization methods.

From the results of single-peak reference functions F1–F7, MSCSO has significant performance advantages, especially in the performance of F1–F4, where the MSCSO algorithm’s convergence curve quickly reaches the optimal value, while other algorithms remain trapped in local optima, with their curves no longer showing a downward trend as iterations increase. For F5, MSCSO also achieved relatively optimal results. However, in F6 and F7, the optimization results of MSCSO are not good, showing only a certain degree of improvement from the original algorithm. In the multimodal benchmark functions F8–F13, MSCSO performs well in the optimization of multimodal complex dimensional functions. In F8–F11, MSCSO outperforms other optimization algorithms in both convergence accuracy and speed, while the other methods exhibit oscillations in their convergence curves. For F12 and F13, MSCSO’s final optimization results are not as good as DO. In the fixed-dimensional multimodal reference functions F14–F23, these test functions have low dimensionality and are more similar to real-world optimization problems. MSCSO shows better convergence accuracy and more stable convergence curve for F20–F23.

Integrating the above analysis, MSCSO demonstrates, in comparison with other swarm intelligence optimization algorithms, such as SCSO, MFO, the SSA, FVIM, the AOA, the SCA, PSO, DO, and SFO, a generally faster convergence speed, higher convergence accuracy, and more stable convergence performance.

### 4.4. Experiment 2: Feature Selection

#### 4.4.1. Feature Selection Dataset Collection and Preprocessing

This study’s feature selection experiments used 15 datasets from the well-established UCI Machine Learning Repository, a resource that spans various application domains and supports the development and evaluation of machine learning algorithms. The selected 15 datasets span a range of data characteristics and complexities, ensuring the broad applicability and representativeness of the experimental results. Table 4 provides detailed information for each dataset, including names, sample count, feature count, and class count.

For datasets containing missing values, numerical missing values are filled by the median and categorical missing values are filled by the mode. The dataset is split into training and test sets with an 80/20 ratio, and the KNN algorithm is applied with K = 5. All algorithms are evaluated under the same experimental conditions to ensure fairness and comparability. Each algorithm uses 30 search agents for 100 iterations. To mitigate the impact of random initialization, each algorithm is independently executed 20 times.

#### 4.4.2. Numerical and Statistical Analysis of Experiment 2

This section assesses MSCSO’s performance in feature selection using various indicators. Table 5, Table 6 and Table 7 display the comparison of MSCSO with other optimization algorithms across different datasets. In order to highlight the best performance of the algorithm, the best values in the table are identified in bold font. To provide a clearer comparison of overall performance, the final rows summarize each algorithm’s average and overall rankings across 15 datasets.

**Figure 3:**
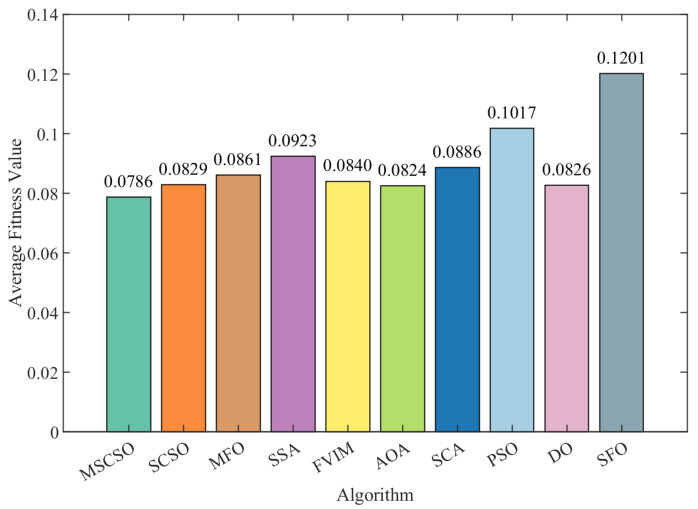
Average fitness across 15 datasets.

Table 5 presents the average fitness and standard deviation for various optimization methods across each dataset. The data in the analysis table shows that MSCSO performs well on most datasets. Of the 15 datasets tested, MSCSO achieved the best average fitness on 14 datasets, accounting for up to 93.33%, showing a significant advantage. The only exception was the M-of-n dataset, where the mean fitness of MSCSO was slightly lower than that of the FVIM optimization algorithm, although this difference was not significant and had a more limited impact on the overall results. Further analysis revealed that MSCSO performed particularly well in comparison to the original SCSO, outperforming SCSO in all datasets. In addition, the overall ranking shows that MSCSO is the top and best performing algorithm, while PSO is the lowest ranking algorithm. The ranking order is MSCSO > AOA > SCSO > DO > FVIM > MFO > SCA > SSA > SFO > PSO. Referring further to Figure 3, which visually shows the average fitness of different optimization algorithms across 15 datasets, it is clear that MSCSO has a lower mean fitness value and a better effect.

**Table 5:** Comparison table of mean fitness and standard deviations between MSCSO and other optimization algorithms in feature selection (Bold indicates better data performance).

Dataset	Measures	MSCSO	SCSO	MFO	SSA	FVIM	AOA	SCA	PSO	DO	SFO
Zoo	Mean	**0.0411**	0.0431	0.0444	0.0476	0.0428	0.0424	0.0483	0.0588	0.0412	0.0701
	Std	0.0064	0.0068	0.0064	0.0064	0.0061	**0.0047**	0.0074	0.0953	0.0062	0.0081
Wine	Mean	**0.0316**	0.0324	0.0339	0.0372	0.0333	0.0318	0.0343	0.0469	0.0329	0.0440
	Std	0.0037	0.0034	0.0044	**0.0025**	0.0042	0.0037	0.0043	0.0964	0.0042	0.0094
Vote	Mean	**0.0826**	0.0903	0.0955	0.1042	0.0906	0.0897	0.0998	0.1152	0.0876	0.1073
	Std	0.0092	0.0083	0.0086	**0.0075**	0.0107	0.0077	0.0115	0.0901	0.0094	0.0164
Lymphography	Mean	**0.1021**	0.1099	0.1137	0.1196	0.1110	0.1089	0.1115	0.1274	0.1065	0.1468
	Std	0.0134	0.0124	0.0108	**0.0080**	0.0111	0.0098	0.0147	0.0886	0.0117	0.0085
HeartEW	Mean	**0.1131**	0.1197	0.1142	0.1320	0.1161	0.1185	0.1273	0.1395	0.1167	0.1553
	Std	0.0112	0.0118	0.0123	0.0106	0.0115	**0.0078**	0.0144	0.0876	0.0097	0.0132
Sonar	Mean	**0.0452**	0.0487	0.0520	0.0532	0.0498	0.0512	0.0514	0.0642	0.0513	0.0611
	Std	0.0034	0.0039	0.0032	0.0032	0.0022	**0.0017**	0.0060	0.0946	0.0031	0.0116
SpectEW	Mean	**0.1493**	0.1580	0.1613	0.1652	0.1622	0.1593	0.1655	0.1721	0.1626	0.1862
	Std	0.0114	0.0092	0.0098	0.0084	0.0090	**0.0075**	0.0182	0.0842	0.0117	0.0103
Lung-Cancer	Mean	**0.0515**	0.0553	0.0583	0.0645	0.0616	0.0516	0.0557	0.0706	0.0548	0.1541
	Std	0.0235	0.0198	0.0219	0.0257	0.0616	**0.0080**	0.0129	0.0959	0.0187	0.0260
BreastEW	Mean	**0.0312**	0.0331	0.0357	0.0372	0.0011	0.0355	0.0358	0.0495	0.0346	0.0379
	Std	0.0026	0.0021	0.0017	**0.0011**	0.0020	0.0014	0.0037	0.0961	0.0016	0.0064
CongressEW	Mean	**0.0315**	0.0349	0.0379	0.0412	0.0348	0.0334	0.0402	0.0567	0.0359	0.0483
	Std	0.0054	0.0067	0.0059	**0.0041**	0.0055	0.0044	0.0059	0.0955	0.0062	0.0112
Clean1	Mean	**0.1200**	0.1272	0.1336	0.1386	0.1315	0.1279	0.1346	0.1493	0.1246	0.1417
	Std	0.0066	0.0062	0.0058	0.0037	0.0049	**0.0035**	0.0140	0.0862	0.0061	0.0113
Exactly	Mean	**0.0635**	0.0690	0.0812	0.1026	0.0662	0.0638	0.0870	0.1005	0.0652	0.2544
	Std	0.0359	0.0424	0.0546	0.0447	0.0412	0.0293	0.0380	0.1015	0.0430	**0.0096**
	Rank	**1**	5	6	9	4	2	7	8	3	10
Exactly2	Mean	**0.1727**	0.1740	0.1770	0.1812	0.1757	0.1739	0.1782	0.1916	0.1740	0.1833
	Std	0.0034	0.0036	0.0040	0.0038	0.0040	**0.0031**	0.0181	0.0818	0.0038	0.0147
M-of-n	Mean	0.0541	0.0561	0.0592	0.0654	**0.0537**	0.0541	0.0654	0.0734	0.0576	0.1169
	Std	0.0148	0.0160	0.0183	0.0175	0.0141	0.0129	0.0158	0.0950	0.0155	**0.0105**
VP	Mean	**0.0895**	0.0915	0.0942	0.0948	0.0940	0.0945	0.0932	0.1101	0.0942	0.0935
	Std	0.0016	0.0007	0.0004	**0.0001**	0.0002	**0.0001**	0.0094	0.0899	0.0002	0.0065
Avg. rank		**1.07**	3.47	5.53	7.73	4.40	3.20	6.40	9.20	3.60	8.93

**Figure 4:**
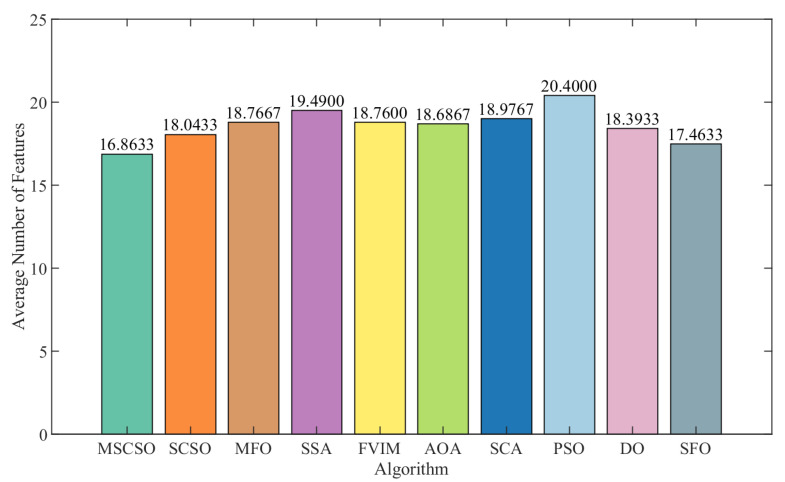
Average number of features across 15 datasets.

Table 6 provides detailed data on the number of features selected by each optimization method. The count of features selected is a crucial index for assessing the effectiveness of optimization methods. From the table data, it is clear that MSCSO selected the least number of features on average in 13 datasets, and only in the SpectEW and Clean1 datasets was the number of features selected lower than that of SFO, ranking second. On the Wine dataset, MSCSO and the AOA each select an average of 3.65 features, while the DO algorithm selects an average of 5.95 features in the Zoo dataset. In summary, MSCSO ranked first in the number of feature selection, SCSO ranked second, and PSO ranked lowest. In terms of standard deviation, MSCSO showed the lowest standard deviation among the nine datasets, indicating excellent stability and robustness; that is, consistent performance over multiple runs. Figure 4 further highlights the advantage of MSCSO in feature selection. The figure illustrates that MSCSO selects the least number of features on average in multiple datasets, which further proves the superiority of MSCSO in the process of feature number selection.

**Table 6:** Comparison table of average feature selection size and standard deviation between MSCSO and other optimization algorithms (Bold indicates better data performance).

Dataset	Measures	MSCSO	SCSO	MFO	SSA	FVIM	AOA	SCA	PSO	DO	SFO
Zoo	Mean	**5.95**	6.2	6.15	7.05	6	6.2	7.1	7.15	**5.95**	8.15
	Std	**0.3940**	0.6156	0.5871	0.7592	0.6489	0.8335	0.5525	0.4894	0.6048	2.1831
Wine	Mean	**3.65**	3.8	3.9	4.55	3.9	**3.65**	4.3	4.25	3.85	5.15
	Std	0.4894	0.6156	**0.3078**	0.6048	0.4472	0.4894	0.5712	0.6387	0.4894	1.0894
Vote	Mean	**4.3**	4.8	4.4	4.95	4.6	4.9	4.75	4.85	4.45	4.8
	Std	**0.6569**	1.1517	0.9403	1.2763	0.9947	1.0208	0.9665	0.8127	0.7592	1.3992
Lymphography	Mean	**8.25**	8.45	8.9	9.45	9.1	9.25	8.5	9.1	8.75	9.15
	Std	1.4824	**0.9987**	1.2937	1.0501	1.0208	1.1180	1.3955	1.0711	1.2085	1.4965
HeartEW	Mean	**4**	4.85	4.25	5.75	4.25	4.95	5.85	6.1	4.95	5.35
	Std	0.8584	1.2680	1.0195	1.4096	**0.8507**	1.7614	1.2680	1.5526	1.5035	1.1821
Sonar	Mean	**25.9**	27.65	29.9	30.15	28.65	29.95	29.85	31	29.15	28.1
	Std	**1.4832**	2.1095	2.2688	2.2308	2.4554	2.0894	2.1588	1.5560	1.8994	2.5526
SpectEW	Mean	10.45	11.4	12.55	12.6	12.4	12.75	13.15	12.15	12.2	**9.7**
	Std	2.2355	2.1619	2.2355	2.6238	2.3486	1.9160	2.1588	2.7961	2.0417	**1.5927**
Lung-Cancer	Mean	**23.9**	26.1	28.2	29.45	27.65	26.9	28.8	30.05	27.25	25.55
	Std	2.5319	2.9182	**1.9084**	2.4810	2.2775	3.1606	3.6935	2.1145	1.9160	3.0517
BreastEW	Mean	**8.85**	9.35	10.1	10.65	9.9	10	10.45	10.95	9.7	10.9
	Std	**0.8127**	**0.8127**	0.9119	1.5313	1.2096	1.1239	1.1910	0.9987	0.9787	1.1653
CongressEW	Mean	**2.65**	3	3.15	4.2	3.15	3.1	4.25	4.45	2.95	4.65
	Std	**0.4894**	0.7947	0.6708	1.0052	0.8127	0.5525	0.6387	0.8870	0.9445	1.6311
Clean1	Mean	89.65	95.8	97	99.7	99.1	95.35	94.5	102.6	95.6	**80.15**
	Std	9.1495	10.2834	8.4043	10.4584	8.9731	8.7856	9.7306	**5.4328**	7.9763	6.8077
Exactly	Mean	**6.05**	6.25	6.5	7.15	6.2	6.25	6.95	6.95	6.1	6.5
	Std	**0.2236**	0.4443	0.5130	0.4894	0.4104	0.4443	0.3940	0.5104	0.3078	2.1643
Exactly2	Mean	**1**	1.25	1.35	1.85	1.25	1.15	2.15	2.2	1.05	2.45
	Std	**0**	0.4443	0.4894	0.7452	0.4443	0.3663	0.5871	0.8944	0.2236	0.8256
M-of-n	Mean	**6.1**	6.15	6.35	6.85	6.15	6.15	6.8	6.9	6.3	7.1
	Std	**0.3078**	0.3663	0.4894	0.6708	0.3663	0.3663	0.6156	0.4472	0.4702	1.6827
VP	Mean	**52.25**	55.6	58.8	58	59.1	59.75	57.25	67.3	57.65	54.25
	Std	**2.2449**	3.6907	3.9815	4.3649	4.9407	4.3271	5.4374	2.9753	6.5154	5.4374
Avg. rank		**1.13**	3.27	5.07	7.33	4.53	5.13	6.00	7.80	3.47	5.47

**Figure 5:**
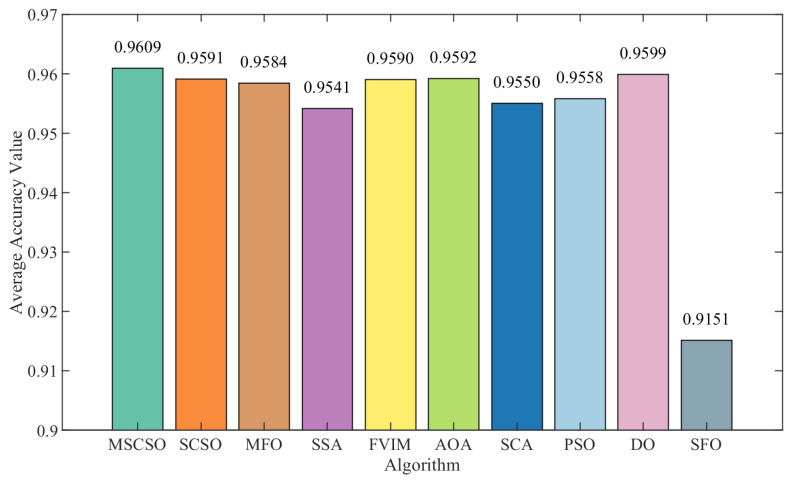
Average accuracy across 15 datasets.

Additionally, to evaluate MSCSO’s effectiveness, the average accuracy of feature subsets selected by various optimization algorithms is compared, with the results presented in Table 7. The analysis showed that MSCSO achieved the highest accuracy across all 15 datasets. In contrast, SCSO and MFO performed well in nine datasets, the AOA, DO, and FVIM performed well in eight datasets, PSO achieved high accuracy in six datasets, the SSA and SCA performed well in five datasets, and SFO performed well only in Elactly2. Overall, MSCSO outperforms other optimization methods in average accuracy, which fully proves that it still has an excellent capability of relevant feature recognition even when the feature subset size is not dominant. For example, in the SpectEW dataset, while SFO selected the lowest average number of features at 9.7, MSCSO achieved a higher accuracy of 0.8991 with a slightly higher number of features at 10.45 compared to SFO’s accuracy of 0.8434. In the Clean1 dataset, SFO selected an average of 80.15 features, while MSCSO selected 89.65 features, with accuracy rates of 0.8979 and 0.9337, respectively. In addition, MSCSO selected the lowest average number of features in some datasets but still achieved the highest accuracy. As a result, MSCSO achieves a better balance between maximizing accuracy and minimizing feature subsets. Figure 5 illustrates the average accuracy across 15 datasets, further highlighting the benefits of MSCSO.

**Table 7:** Comparison table of average accuracy and standard deviations of MSCSO and other optimization algorithms (Bold indicates better data performance).

Dataset	Measures	MSCSO	SCSO	MFO	SSA	FVIM	AOA	SCA	PSO	DO	SFO
Zoo	Mean	**1**	**1**	**1**	**1**	**1**	**1**	**1**	**1**	**1**	0.9800
	Std	**0**	**0**	**0**	**0**	**0**	**0**	**0**	**0**	**0**	0.0251
Wine	Mean	**1**	**1**	**1**	**1**	**1**	**1**	**1**	**1**	**1**	0.9971
	Std	**0**	**0**	**0**	**0**	**0**	**0**	**0**	**0**	**0**	0.0088
Vote	Mean	**0.9467**	0.9425	0.9333	0.9275	0.9433	0.9417	0.9275	0.9292	0.9442	0.9167
	Std	**0.0068**	0.0101	0.0108	0.0135	0.0100	0.0086	0.0124	0.0142	0.0082	0.0162
Lymphography	Mean	**0.9534**	0.9431	0.9414	0.9328	0.9466	0.9483	0.9362	0.9328	0.9483	0.8948
	Std	0.0169	0.0169	0.0162	**0.0077**	0.0176	0.0177	0.0126	**0.0077**	0.0177	0.0136
HeartEW	Mean	**0.9241**	0.9222	0.9213	0.9130	0.9222	0.9231	0.9148	0.9194	0.9231	0.8750
	Std	**0.0057**	0.0076	0.0082	0.0087	0.0076	0.0068	0.0093	0.0091	0.0068	0.0158
Sonar	Mean	**1**	**1**	**1**	**1**	**1**	**1**	**1**	**1**	**1**	0.9866
	Std	**0**	**0**	**0**	**0**	**0**	**0**	**0**	**0**	**0**	0.0124
SpectEW	Mean	**0.8991**	0.8934	0.8981	0.8887	0.8925	0.8953	0.8915	0.8915	0.8962	0.8434
	Std	**0.0111**	0.0176	0.0128	0.0161	0.0151	0.0156	0.0148	0.0161	0.0115	0.0195
Lung-Cancer	Mean	**1**	**1**	**1**	**1**	**1**	**1**	**1**	**1**	**1**	0.8833
	Std	**0**	**0**	**0**	**0**	**0**	**0**	**0**	**0**	**0**	0.0784
BreastEW	Mean	**1**	**1**	**1**	0.9996	0.9991	0.9991	0.9991	0.9996	0.9996	0.9991
	Std	**0**	**0**	**0**	0.0020	0.0027	0.0027	0.0027	0.0020	0.0020	0.0027
CongressEW	Mean	**0.9884**	**0.9884**	**0.9884**	0.9878	**0.9884**	**0.9884**	0.9878	0.9866	**0.9884**	0.9802
	Std	**0**	**0**	**0**	0.0046	**0**	**0**	0.0026	0.0043	**0**	0.0114
Clean1	Mean	**0.9337**	0.9305	0.9258	0.9174	0.9263	0.9258	0.9147	0.9195	0.9332	0.8979
	Std	0.0103	0.0093	0.111	0.0104	0.0084	0.0093	0.0090	**0.0052**	0.0129	0.0128
Exactly	Mean	**1**	**1**	**1**	0.9828	0.9995	**1**	0.9905	0.9915	**1**	0.7748
	Std	**0**	**0**	**0**	0.0212	0.0022	**0**	0.0150	0.0171	**0**	0.0983
Exactly2	Mean	**0.82**	**0.82**	**0.82**	**0.82**	**0.82**	**0.82**	**0.82**	0.82	**0.82**	**0.82**
	Std	**0**	**0**	**0**	**0**	**0**	**0**	**0**	0.0031	**0**	**0**
M-of-n	Mean	**1**	**1**	**1**	0.9975	**1**	**1**	0.9983	0.9993	**1**	0.9323
	Std	**0**	**0**	**0**	0.0062	**0**	**0**	0.0054	0.0034	**0**	0.0513
VP	Mean	**0.9474**	0.9470	0.9470	0.9451	0.9470	0.9470	0.9451	**0.9474**	0.9459	0.9451
	Std	**0**	0.0017	0.0017	0.0035	0.0017	0.0017	0.0035	**0**	0.0031	0.0035
Avg. rank		**1**	2	2.2	3.93	2.2	2.07	3.67	3.2	1.6	4.87

#### 4.4.3. Graphical Analysis of Experiment 2

**Figure 6:**
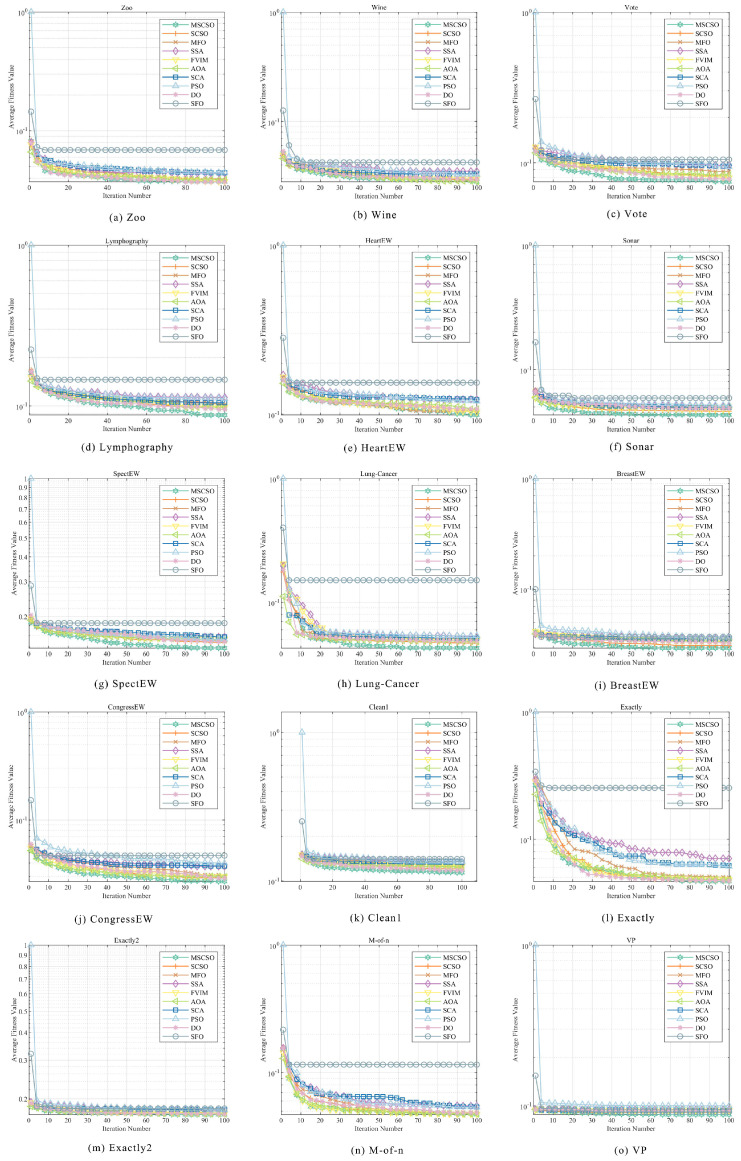
Comparison of convergence curves of MSCSO feature selection with other optimization algorithms.

Figure 6 shows the convergence curve in the feature selection task. By analyzing the convergence curve, the convergence speed and final convergence effect of different algorithms can be evaluated. The figure clearly shows that MSCSO achieved the lowest mean fitness values on eight datasets, which were Vote, Lymphography, Sonar, SpectEW, Lung-Cancer, BreastEW, CongressEW, and Clean1. This indicates that the algorithm excels with these datasets. Further combining the results of Table 5, MSCSO also performed well on the Zoo, Wine, HeartEW, Exactly, Exactly2, and VP datasets, only slightly worse than FVIM optimization on the M-of-n dataset. In addition, it is worth noting that MSCSO performed better on mid-dimensional datasets (such as Sonar, Lung-Cancer, and BreastEW) than it did on low-dimensional datasets and that the performance gap between MSCSO and other algorithms was greater in mid-dimensional datasets than in low-dimensional datasets. These results show that the MSCSO algorithm has significant advantages in feature selection tasks, especially on medium-dimensional datasets.

## 5. Conclusions and Future Directions

The MSCSO algorithm proposed in this study has demonstrated significant advantages in global optimization and feature selection tasks. In the CEC2005 benchmark tests, MSCSO excelled on 65.2% of the test functions, achieving the best average fitness and outperforming nine common heuristics in terms of both convergence speed and precision. The Wilcoxon test further confirmed the significant performance differences between MSCSO and other algorithms. During feature selection, through experiments on 15 UCI datasets, MSCSO achieved the best average fitness in 93.3% of the datasets and selected the least number of features in 86.7% of the datasets. Moreover, MSCSO also achieved the highest classification accuracy across all datasets. These results indicate that MSCSO has shown excellent effectiveness in global optimization and feature selection tasks.

In the future, the further development of MSCSO can be carried out in the following directions: firstly, MSCSO can be combined with other optimization algorithms to form a hybrid algorithm to give full play to their respective advantages. Secondly, exploring a variety of binarization methods may further improve the efficiency and accuracy of feature selection. Finally, the application potential of MSCSO is not limited to the current experimental field and can also be extended to image processing, bioinformatics, financial data analysis, and other fields to verify its applicability and effectiveness in a wider range of scenarios.

## Figures and Tables

**Table 1:** Parameter information of each algorithm.

Algorithm	Parameter
All	Global optimization parameters: Population size = 30, Maximum iterations = 1000, Run count = 30 Feature selection parameters: Population size = 30, Maximum iterations = 100, Run count = 20
MSCSO	k = 0.75, *p* = [1:360], S = 2
SCSO	*p* = [1:360], S = 2
MFO	b = 1
SSA	-
FVIM	alpha = 1.5
AOA	MOP_Max = 1, MOP_Min = 0.2, Alpha = 5, Mu = 0.499
SCA	a = 2
PSO	w = 0.85, c1 = 1.2, c2 = 1.2
DO	-
SFO	PD = 2/3

**Table 2:** Comparison of mean fitness and standard deviation between MSCSO and existing optimization algorithms in CEC2005 test function (Bold indicates better data performance).

Function	Measures	MSCSO	SCSO	MFO	SSA	FVIM	AOA	SCA	PSO	DO	SFO
F1	Mean	**0**	1.6732 × 10^−22^	6.6835	1.2217 × 10^−8^	7.8252 × 10^−55^	1.0115 × 10^−18^	5.011 × 10^−2^	8.8258 × 10^−7^	1.2027 × 10^−8^	5.9286 × 10^−19^
	Std	**0**	**0**	2.5366	2.8644 × 10^−9^	2.3666 × 10^−54^	5.5403 × 10^−18^	1.2384 × 10^−1^	1.2243 × 10^−6^	8.8765 × 10^−9^	1.2626 × 10^−18^
F2	Mean	**0**	1.2274 × 10^−11^	3.3333	8.7898 × 10^−1^	6.0333 × 10^−32^	**0**	8.8029 × 10^−5^	3.4267 × 10^−1^	7.9067 × 10^−5^	2.9971 × 10^−9^
	Std	**0**	6.5811 × 10^−11^	2.0228	8.4123 × 10^−1^	7.6058 × 10^−32^	**0**	4.1444 × 10^−4^	1.8244	3.3934 × 10^−5^	2.3602 × 10^−9^
F3	Mean	**0**	7.1882 × 10^−19^	1.4705	2.6345	2.1786 × 10^−6^	2.6499 × 10^−3^	3.3952 × 10^3^	2.5677	1.0250	1.2079 × 10^−16^
	Std	**0**	**0**	1.0837	2.1461	7.2399 × 10^−6^	7.6668 × 10^−3^	3.4423 × 10^3^	1.1124	1.2048	2.1024 × 10^−16^
F4	Mean	**0**	9.6592 × 10^−10^	6.8430	8.0524	7.1856 × 10^−2^	1.6863 × 10^−2^	1.9320 × 10^1^	3.8051	9.6583 × 10^−2^	2.0926 × 10^−10^
	Std	**0**	4.8151 × 10^−10^	8.5460	3.5330	3.1181 × 10^−2^	2.1198 × 10^−2^	1.3087 × 10^1^	1.3945	5.1918 × 10^−2^	1.8565 × 10^−10^
F5	Mean	**1.3318**	2.8212 × 10^1^	6.8812	1.8062	3.0309 × 10^1^	2.8215 × 10^1^	5.1516 × 10^2^	9.4887	2.8709 × 10^1^	2.8706 × 10^1^
	Std	1.2855	6.9124 × 10^−1^	2.2651	3.6087	1.1645 × 10^1^	4.3180 × 10^−1^	1.6177 × 10^3^	1.1385	1.6104 × 10^1^	**1.4421 × 10^−3^**
F6	Mean	1.67	1.82	2.3367	**1.2538 × 10^−8^**	3.5837	2.7693	4.7484	9.4045 × 10^−7^	9.1274 × 10^−7^	3.1592
	Std	6.3222	6.1323 × 10^−1^	6.8034	**2.4312 × 10^−9^**	8.3087 × 10^−1^	2.4581 × 10^−1^	8.6016 × 10^−1^	1.0517 × 10^−6^	4.1544 × 10^−7^	2.2552
F7	Mean	6.1529	9.1979 × 10^−5^	4.0113	1.0180 × 10^−1^	7.4159 × 10^−3^	**3.9189 × 10^−5^**	2.9036 × 10^−2^	2.4734 × 10^−2^	9.3052 × 10^−3^	8.1435 × 10^−5^
	Std	6.1166	1.1144 × 10^−4^	8.6251	3.7415 × 10^−2^	3.1360 × 10^−3^	**4.5493 × 10^−5^**	2.2559 × 10^−2^	7.8311 × 10^−3^	4.9979 × 10^−3^	6.9022 × 10^−5^
F8	Mean	**−1.203**	−6.9303	−8.9559	−7.3619	−5.3718	−5.8265	−3.8623	−7.6221	−8.2970	−3.9342
	Std	1.1679	8.6264 × 10^2^	9.3253	6.9246	1.1014 × 10^3^	3.7253 × 10^2^	**2.8576 × 10^2^**	7.3327	6.2155 × 10^2^	1.1031 × 10^3^
F9	Mean	**0**	**0**	1.7763	5.8006	3.5712 × 10^1^	**0**	1.6014 × 10^1^	4.8457	1.5862 × 10^1^	**0**
	Std	**0**	**0**	4.3103	2.2665	9.3304	**0**	1.9496 × 10^1^	1.3825	1.1639 × 10^1^	**0**
F10	Mean	**4.4409 × 10^−1^**	**4.4409 × 10^−1^**	1.2452	2.0840	8.1416 × 10^−15^	**4.4409 × 10^−1^**	1.4426 × 10^1^	4.5028 × 10^−1^	2.4648 × 10^−5^	5.2770 × 10^−10^
	Std	**0**	**0**	8.5861	8.4850 × 10^−1^	1.8853 × 10^−15^	**0**	8.1441	6.5171 × 10^−1^	1.1472 × 10^−5^	5.3965 × 10^−10^
F11	Mean	**0**	**0**	1.5158	1.1401 × 10^−2^	5.5451 × 10^−3^	9.9232 × 10^−2^	2.7640 × 10^−1^	1.9719 × 10^−2^	1.5936 × 10^−2^	**0**
	Std	**0**	**0**	3.4452	1.1605 × 10^−2^	8.8110 × 10^−3^	8.2692 × 10^−2^	2.8250 × 10^−1^	2.5921 × 10^−2^	1.6144 × 10^−2^	**0**
F12	Mean	2.6045	7.3425 × 10^−2^	1.5387	4.8849 × 10^1^	8.1274 × 10^−1^	4.0717 × 10^−1^	6.5974 × 10^2^	8.5519 × 10^−2^	**6.9630 × 10^−8^**	2.7687 × 10^−1^
	Std	1.3674	2.9459 × 10^−2^	4.6668	3.0881	6.0639 × 10^−1^	4.6520 × 10^−2^	3.5664 × 10^3^	2.0937 × 10^−1^	**3.6371 × 10^−8^**	3.8328 × 10^−1^
F13	Mean	4.7895	2.4395	1.3669	1.6889	2.2887	2.7819	4.5932 × 10^2^	6.2330 × 10^−3^	**1.0343 × 10^−6^**	1.0019 × 10^−2^
	Std	1.0445	4.2140 × 10^−1^	7.4867	6.2236	3.9923 × 10^−1^	1.2718 × 10^−1^	2.4504 × 10^3^	1.1425 × 10^−2^	**3.7267 × 10^−7^**	3.2748 × 10^−2^
F14	Mean	1.0311	6.0842	2.3483	**9.98 × 10^−1^**	9.1841	1.0218 × 10^1^	1.4618	**9.98 × 10^−1^**	**9.98 × 10^−1^**	8.1515
	Std	1.8148	4.5054	1.9251	2.6562 × 10^−16^	4.6576	3.1487	8.5309 × 10^−1^	**4.1233 × 10^−17^**	4.9736 × 10^−16^	4.8078
F15	Mean	**3.1987**	4.0878 × 10^−4^	1.7980 × 10^−3^	8.3495 × 10^−4^	5.0951 × 10^−3^	2.2018 × 10^−2^	8.5939 × 10^−4^	3.6175 × 10^−3^	1.1016 × 10^−3^	1.8113 × 10^−2^
	Std	**3.9231**	2.7855 × 10^−4^	3.5397 × 10^−3^	2.8094 × 10^−4^	8.6189 × 10^−3^	3.5493 × 10^−2^	3.5571 × 10^−4^	1.1204 × 10^−2^	3.6516 × 10^−3^	3.0145 × 10^−2^
F16	Mean	**−1.031628**	**−1.0316284**	**−1.0316284**	**−1.0316284**	−1.0316282	−1.0316283	−1.0316002	**−1.0316284**	**−1.0316284**	−1.0316282
	Std	4.0375 × 10^−1^	2.4429 × 10^−10^	**6.7752 × 10^−16^**	7.2408 × 10^−15^	8.8799 × 10^−7^	1.0438 × 10^−7^	2.7392 × 10^−5^	**6.7752 × 10^−16^**	8.2357 × 10^−14^	1.0214 × 10^−6^
F17	Mean	0.3978873	0.39788737	**0.39788736**	**0.39788736**	0.39788755	0.40560883	0.39892979	**0.39788736**	**0.39788736**	0.39791132
	Std	5.1770	2.0828 × 10^−8^	**0**	5.2723 × 10^−15^	4.3609 × 10^−7^	7.5248 × 10^−3^	9.4806 × 10^−4^	**0**	3.4351 × 10^−12^	1.0370 × 10^−4^
F18	Mean	3.0000007	3.00000123	**3**	**3**	3.00003310	10.9652648	3.00003931	**3**	3.00000000	12.0000119
	Std	1.5252	1.8880 × 10^−6^	1.3297 × 10^−15^	1.3386 × 10^−13^	5.1040 × 10^−5^	1.2387 × 10^1^	8.7638 × 10^−5^	**8.8049 × 10^−16^**	2.2231 × 10^−9^	2.4901 × 10^1^
F19	Mean	3.8627805	−3.8616140	**−3.8627821**	**−3.8627821**	−3.8627257	−3.8536551	−3.8550555	**−3.8627821**	−3.8627821	−3.8035099
	Std	2.3680 × 10^−6^	2.7517 × 10^−3^	2.7101 × 10^−15^	5.2138 × 10^−14^	6.4765 × 10^−5^	2.6938 × 10^−3^	1.8405 × 10^−3^	**2.6823 × 10^−15^**	2.3382 × 10^−8^	1.9563 × 10^−1^
F20	Mean	**−3.322**	−3.2477	−3.1982	−3.2238	−3.2821	−3.0976	−2.8989	−3.2679	−3.2744	−2.9596
	Std	**2.1588 × 10^−6^**	1.1059 × 10^−1^	5.7419 × 10^−2^	5.0081 × 10^−2^	5.7438 × 10^−2^	7.5351 × 10^−2^	3.0548 × 10^−1^	6.9818 × 10^−2^	5.9241 × 10^−2^	1.9696 × 10^−1^
F21	Mean	**−9.983**	−5.0860	−6.3886	−7.3946	−7.1978	−3.7087	−2.2721	−5.7286	−6.0454	−5.7652
	Std	**9.3075 × 10^−1^**	1.2230	3.4639	3.3091	3.2043	1.1244	1.9682	3.3243	3.3225	2.8908
F22	Mean	**−9.694**	−6.8608	−8.7772	−9.5422	−9.5982	−4.2464	−3.1372	−6.5563	−7.3459	−5.8515
	Std	1.8377	2.5475	3.0521	2.2747	2.2776	**1.6229**	1.7211	3.5019	3.6572	2.9746
F23	Mean	**−9.996**	−6.4471	−7.4247	−8.2498	−9.7547	−4.2953	−3.8125	−7.6379	−6.7571	−6.0959
	Std	1.6501	3.1867	3.6601	3.5821	2.3855	**1.1388**	1.9384	3.6788	3.7216	3.1840
Avg. rank		**1.57**	3.61	6.22	4.52	4.48	5.65	7.70	4.65	3.61	5.39

**Table 3:** Comparison of Wilcoxon test *p*-values between MSCSO and existing optimization algorithms (Underline indicates no significant difference between MSCSO and the algorithm).

Function	SCSO	MFO	SSA	FVIM	AOA	SCA	PSO	DO	SFO
F1	1.21 × 10^−12^	1.21 × 10^−12^	1.21 × 10^−12^	4.57 × 10^−12^	1.21 × 10^−12^	1.21 × 10^−12^	1.21 × 10^−12^	1.21 × 10^−12^	1.21 × 10^−12^
F2	1.21 × 10^−12^	1.21 × 10^−12^	1.21 × 10^−12^	1.00	1.21 × 10^−12^	1.21 × 10^−12^	1.21 × 10^−12^	1.21 × 10^−12^	1.21 × 10^−12^
F3	1.21 × 10^−12^	1.21 × 10^−12^	1.21 × 10^−12^	1.21 × 10^−12^	1.21 × 10^−12^	1.21 × 10^−12^	1.21 × 10^−12^	1.21 × 10^−12^	1.21 × 10^−12^
F4	1.21 × 10^−12^	1.21 × 10^−12^	1.21 × 10^−12^	1.21 × 10^−12^	1.21 × 10^−12^	1.21 × 10^−12^	1.21 × 10^−12^	1.21 × 10^−12^	1.21 × 10^−12^
F5	1.19 × 10^−6^	3.02 × 10^−11^	2.57 × 10^−7^	1.29 × 10^−6^	4.08 × 10^−5^	5.49 × 10^−11^	1.43 × 10^−5^	0.074827	1.07 × 10^−7^
F6	3.02 × 10^−11^	5.11 × 10^−1^	3.02 × 10^−11^	3.02 × 10^−11^	3.02 × 10^−11^	3.02 × 10^−11^	3.02 × 10^−11^	3.02 × 10^−11^	6.70 × 10^−11^
F7	2.84 × 10^−1^	3.02 × 10^−11^	3.02 × 10^−11^	3.02 × 10^−11^	3.92 × 10^−2^	3.02 × 10^−11^	3.02 × 10^−11^	3.02 × 10^−11^	0.22257
F8	3.02 × 10^−11^	3.82 × 10^−9^	3.02 × 10^−11^	3.02 × 10^−11^	3.02 × 10^−11^	3.02 × 10^−11^	6.70 × 10^−11^	8.99 × 10^−11^	3.02 × 10^−11^
F9	1.00	1.21 × 10^−12^	1.21 × 10^−12^	1.21 × 10^−12^	1.00	1.21 × 10^−12^	1.21 × 10^−12^	1.21 × 10^−12^	1.00
F10	1.00	1.21 × 10^−12^	1.21 × 10^−12^	6.13 × 10^−14^	1.00	1.21 × 10^−12^	1.21 × 10^−12^	1.21 × 10^−12^	1.21 × 10^−12^
F11	1.00	1.21 × 10^−12^	1.21 × 10^−12^	1.37 × 10^−3^	1.21 × 10^−12^	1.21 × 10^−12^	1.21 × 10^−12^	1.21 × 10^−12^	1.00
F12	3.02 × 10^−11^	6.07 × 10^−11^	3.02 × 10^−11^	3.02 × 10^−11^	3.02 × 10^−11^	3.02 × 10^−11^	0.61001	3.02 × 10^−11^	8.15 × 10^−11^
F13	3.02 × 10^−11^	2.32 × 10^−6^	9.94 × 10^−1^	3.02 × 10^−11^	3.02 × 10^−11^	3.02 × 10^−11^	0.0072884	3.02 × 10^−11^	0.0076171
F14	8.84 × 10^−7^	9.47 × 10^−1^	1.41 × 10^−11^	4.07 × 10^−11^	3.02 × 10^−11^	2.87 × 10^−10^	1.72 × 10^−12^	1.44 × 10^−10^	4.97 × 10^−11^
F15	2.32 × 10^−2^	3.01 × 10^−11^	8.99 × 10^−11^	8.15 × 10^−5^	1.33 × 10^−10^	6.70 × 10^−11^	0.52014	0.12967	1.61 × 10^−10^
F16	2.17 × 10^−1^	1.21 × 10^−12^	2.98 × 10^−11^	6.01 × 10^−8^	3.02 × 10^−11^	3.02 × 10^−11^	1.21 × 10^−12^	1.33 × 10^−10^	2.23 × 10^−9^
F17	8.53 × 10^−1^	1.21 × 10^−12^	2.70 × 10^−11^	8.84 × 10^−7^	3.02 × 10^−11^	3.02 × 10^−11^	1.21 × 10^−12^	3.02 × 10^−11^	9.53 × 10^−7^
F18	2.81 × 10^−2^	1.27 × 10^−11^	3.02 × 10^−11^	2.37 × 10^−10^	4.12 × 10^−1^	1.16 × 10^−7^	7.87 × 10^−12^	1.17 × 10^−9^	0.021506
F19	2.61 × 10^−2^	1.21 × 10^−12^	3.01 × 10^−11^	1.25 × 10^−7^	3.02 × 10^−11^	3.02 × 10^−11^	2.36 × 10^−12^	7.77 × 10^−9^	1.73 × 10^−7^
F20	9.12 × 10^−1^	9.92 × 10^−7^	6.77 × 10^−5^	2.25 × 10^−4^	3.02 × 10^−11^	3.02 × 10^−11^	0.17902	0.18577	3.02 × 10^−11^
F21	1.78 × 10^−10^	4.27 × 10^−1^	3.33 × 10^−1^	3.82 × 10^−9^	4.08 × 10^−11^	3.69 × 10^−11^	0.034625	0.099258	7.39 × 10^−11^
F22	6.97 × 10^−3^	2.00 × 10^−4^	6.05 × 10^−7^	5.87 × 10^−4^	3.16 × 10^−10^	9.92 × 10^−11^	0.33154	0.26433	9.76 × 10^−10^
F23	6.10 × 10^−3^	3.52 × 10^−1^	6.97 × 10^−3^	9.21 × 10^−5^	2.37 × 10^−10^	9.92 × 10^−11^	0.14395	0.83026	6.12 × 10^−10^

**Table 4:** Feature selection dataset details.

Dataset	Feature Count	Sample Count	Classes
Zoo	16	101	7
Wine	13	178	3
Vote	16	300	2
Lymphography	18	148	4
HeartEW	13	270	2
Sonar	60	208	2
SpectEW	22	267	2
Lung-Cancer	56	32	3
BreastEW	30	568	2
CongressEW	16	434	2
Clean1	166	476	2
Exactly	13	1000	2
Exactly2	13	1000	2
M-of-n	13	1000	2
VP	128	669	3

## Data Availability

The authors declare that they have no known competing financial interests or personal relationships that could have appeared to influence the work reported in this paper.

## Supplementary Materials

The following supporting information can be downloaded at: https://www.mdpi.com/article/10.3390/biomimetics9110701/s1, Table S1: CEC2005 test function details.

## References

[1] Zebari R., Abdulazeez A., Zeebaree D., Zebari D., Saeed J. (2020). A comprehensive review of dimensionality reduction techniques for feature selection and feature extraction. J. Appl. Sci. Technol. Trends.

[2] Houssein E.H., Oliva D., Celik E., Emam M.M., Ghoniem R.M. (2023). Boosted sooty tern optimization algorithm for global optimization and feature selection. Expert Syst. Appl..

[3] Chhabra A., Hussien A.G., Hashim F.A. (2023). Improved bald eagle search algorithm for global optimization and feature selection. Alex. Eng. J..

[4] He J., Qu L., Wang P., Li Z. (2024). An oscillatory particle swarm optimization feature selection algorithm for hybrid data based on mutual information entropy. Appl. Soft Comput..

[5] Sampson J.R. (1976). Adaptation in Natural and Artificial Systems (John H. Holland).

[6] Storn R., Price K. (1997). Differential evolution–a simple and efficient heuristic for global optimization over continuous spaces. J. Glob. Optim..

[7] Huning A. (1976). Evolutionsstrategie. Optimierung Technischer Systeme Nach Prinzipien der Biologischen Evolution.

[8] Gandomi A.H., Alavi A.H. (2012). Krill herd: A new bio-inspired optimization algorithm. Commun. Nonlinear Sci. Numer. Simul..

[9] Hashim F.A., Houssein E.H., Hussain K., Mabrouk M.S., Al-Atabany W. (2022). Honey badger algorithm: New metaheuristic algorithm for solving optimization problems. Math. Comput. Simul..

[10] Mohammed H., Rashid T. (2023). Fox: A fox-inspired optimization algorithm. Appl. Intell..

[11] Kirkpatrick S., Gelatt C.D., Vecchi M.P. (1983). Optimization by simulated annealing. Science.

[12] Talatahari S., Azizi M., Tolouei M., Talatahari B., Sareh P. (2021). Crystal structure algorithm (crystal): A metaheuristic optimization method. IEEE Access.

[13] Rashedi E., Nezamabadi-Pour H., Saryazdi S. (2009). Gsa: A gravitational search algorithm. Inf. Sci..

[14] Gao Y., Zhang J., Wang Y., Wang J., Qin L. (2024). Love evolution algorithm: A stimulus–value–role theory-inspired evolutionary algorithm for global optimization. J. Supercomput..

[15] Binu D., Kariyappa B. (2018). Ridenn: A new rider optimization algorithm-based neural network for fault diagnosis in analog circuits. IEEE Trans. Instrum. Meas..

[16] Nemati M., Zandi Y., Agdas A.S. (2024). Application of a novel meta-heuristic algorithm inspired by stadium spectators in global optimization problems. Sci. Rep..

[17] Bai J., Li Y., Zheng M., Khatir S., Benaissa B., Abualigah L., Wahab M.A. (2023). A sinh cosh optimizer. Knowl.-Based Syst..

[18] Zhao S., Zhang T., Cai L., Yang R. (2024). Triangulation topology aggregation optimizer: A novel mathematics-based meta-heuristic algorithm for continuous optimization and engineering applications. Expert Syst. Appl..

[19] Abdel-Basset M., El-Shahat D., Jameel M., Abouhawwash M. (2023). Exponential distribution optimizer (edo): A novel math-inspired algorithm for global optimization and engineering problems. Artif. Intell. Rev..

[20] Wolpert D.H., Macready W.G. (1997). No free lunch theorems for optimization. IEEE Trans. Evol. Comput..

[21] Seyyedabbasi A., Kiani F. (2023). Sand cat swarm optimization: A nature inspired algorithm to solve global optimization problems. Eng. Comput..

[22] Karami S., Saberi-Movahed F., Tiwari P., Marttinen P., Vahdati S. (2023). Unsupervised feature selection based on variance–covariance subspace distance. Neural Netw..

[23] Cheng J., Sun J., Yao K., Xu M., Cao Y. (2022). A variable selection method based on mutual information and variance inflation factor. Spectrochim. Acta Part A Mol. Biomol. Spectrosc..

[24] Shafizadeh-Moghadam H., Minaei M., Pontius R.G., Asghari A., Dadashpoor H. (2021). Integrating a forward feature selection algorithm, random forest, and cellular automata to extrapolate urban growth in the Tehran-Karaj region of Iran. Comput. Environ. Urban Syst..

[25] Awad M., Fraihat S. (2023). Recursive feature elimination with cross-validation with decision tree: Feature selection method for machine learning-based intrusion detection systems. J. Sens. Actuator Netw..

[26] Afrin S., Shamrat F.J.M., Nibir T.I., Muntasim M.F., Moharram M.S., Imran M., Abdulla M. (2021). Supervised machine learning based liver disease prediction approach with lasso feature selection. Bull. Electr. Eng. Inform..

[27] Malik A., Jamei M., Ali M., Prasad R., Karbasi M., Yaseen Z.M. (2022). Multi-step daily forecasting of reference evapotranspiration for different climates of India: A modern multivariate complementary technique reinforced with ridge regression feature selection. Agric. Water Manag..

[28] Li X.D., Wang J.S., Liu Y., Song H.M., Wang Y.C., Hou J.N., Zhang M., Hao W.K. (2024). Classification feature selection and dimensionality reduction based on logical binary sine-cosine function arithmetic optimization algorithm. Egypt. Inform. J..

[29] Qiu Y., Li R., Zhang X. (2024). Simultaneous svm parameters and feature selection optimization based on improved slime mould algorithm. IEEE Access.

[30] Hussien R.M., Abohany A.A., Abd El-Mageed A.A., Hosny K.M. (2024). Improved binary meerkat optimization algorithm for efficient feature selection of supervised learning classification. Knowl. Based Syst..

[31] Hashim F.A., Houssein E.H., Mostafa R.R., Hussien A.G., Helmy F. (2023). An efficient adaptive-mutated coati optimization algorithm for feature selection and global optimization. Alex. Eng. J..

[32] Pan H., Chen S., Xiong H. (2023). A high-dimensional feature selection method based on modified gray wolf optimization. Appl. Soft Comput..

[33] Peng L., Cai Z., Heidari A.A., Zhang L., Chen H. (2023). Hierarchical Harris Hawks optimizer for feature selection. J. Adv. Res..

[34] Moosavi S.K.R., Saadat A., Abaid Z., Ni W., Li K., Guizani M. (2024). Feature selection based on dataset variance optimization using hybrid sine cosine–firehawk algorithm (hscfha). Future Gener. Comput. Syst..

[35] Kwakye B.D., Li Y., Mohamed H.H., Baidoo E., Asenso T.Q. (2024). Particle guided metaheuristic algorithm for global optimization and feature selection problems. Expert Syst. Appl..

[36] Abdelhamid A.A., El-Kenawy E.S.M., Ibrahim A., Eid M.M., Khafaga D.S., Alhussan A.A., Mirjalili S., Khodadadi N., Lim W.H., Shams M.Y. (2023). Innovative feature selection method based on hybrid sine cosine and dipper throated optimization algorithms. IEEE Access.

[37] Ragab M. (2024). Hybrid firefly particle swarm optimisation algorithm for feature selection problems. Expert Syst..

[38] Alkanhel R., El-kenawy E.S.M., Abdelhamid A.A., Ibrahim A., Alohali M.A., Abotaleb M., Khafaga D.S. (2023). Network intrusion detection based on feature selection and hybrid metaheuristic optimization. Comput. Mater. Contin..

[39] Mirjalili S. (2015). Moth-flame optimization algorithm: A novel nature-inspired heuristic paradigm. Knowl.-Based Syst..

[40] Mirjalili S., Gandomi A.H., Mirjalili S.Z., Saremi S., Faris H., Mirjalili S.M. (2017). Salp swarm algorithm: A bio-inspired optimizer for engineering design problems. Adv. Eng. Softw..

[41] Fakhouri H.N., Awaysheh F.M., Alawadi S., Alkhalaileh M., Hamad F. (2024). Four vector intelligent metaheuristic for data optimization. Computing.

[42] Abualigah L., Diabat A., Mirjalili S., Abd Elaziz M., Gandomi A.H. (2021). The arithmetic optimization algorithm. Comput. Methods Appl. Mech. Eng..

[43] Mirjalili S. (2016). Sca: A sine cosine algorithm for solving optimization problems. Knowl. Based Syst..

[44] Kennedy J., Eberhart R. (1995). Particle swarm optimization. Proceedings of the ICNN’95-International Conference on Neural Networks.

[45] Zhao S., Zhang T., Ma S., Chen M. (2022). Dandelion optimizer: A nature-inspired metaheuristic algorithm for engineering applications. Eng. Appl. Artif. Intell..

[46] Shadravan S., Naji H.R., Bardsiri V.K. (2019). The sailfish optimizer: A novel nature-inspired metaheuristic algorithm for solving constrained engineering optimization problems. Eng. Appl. Artif. Intell..

