# Supplementary material for: A Novel Adaptive Sand Cat Swarm Optimization Algorithm for Feature Selection and Global Optimization
Source: Biomimetics (Basel). 2024 Nov 15;9(11):701. doi: 10.3390/biomimetics9110701 (PMC11591711; doi:10.3390/biomimetics9110701)
Supplement: Supplementary file 1 [file biomimetics-09-00701-s001.zip › biomimetics-3276748-supplementary.pdf]

biomimetics

MDPI

Supplementary Materials

A Novel Adaptive Sand Cat Swarm Optimization Algorithm for Feature Selection and Global Optimization

Ruru Liu [1], Rencheng Fang [1], Tao Zeng [1], Hongmei Fei [1], Quan Qi [1], Pengxiang Zuo [2], Liping Xu [3] and Wei Liu [2,*]

[1] College of Information Science and Technology, Shihezi University, Shihezi 832000, China;
[2] College of Medicine, Shihezi University, Shihezi 832000, China
[3] College of Science, Shihezi University, Shihezi 832000, China
* Correspondence: weiliu@shzu.edu.cn

Table S1. CEC2005 test function details.

Formula	Dim	Range	F_{min}
$F_1(x)=\sum_{i=1}^{n} x_i^2$	30	[-100,100]	0
$F_2(x)=\sum_{i=1}^{n} \lvert x_i \rvert+\prod_{i=1}^{n} \lvert x_i \rvert$	30	[-10,10]	0
$F_3(x)=\sum_{i=1}^{n}(\sum_{j=1}^{i} x_j)^2$	30	[-100,100]	0
$F_4(x)=\max\{\lvert x_i \rvert : 1 \le i \le n\}$	30	[-100,100]	0
$F_5(x)=\sum_{i=1}^{n-1}[100(x_{i+1}-x_i^2)^2+(x_i-1)^2]$	30	[-30,30]	0
$F_6(x)=\sum_{i=1}^{n}(x_i+5)^2$	30	[-100,100]	0
$F_7(x)=\sum_{i=1}^{n} i \times x_i^4 + random[0,1)$	30	[-1.28,1.28]	0
$F_8(x)=\sum_{i=1}^{n} -x_i \sin(\sqrt{\lvert x_i \rvert})$	30	[-500,500]	-12569.5
$F_9(x)=\sum_{i=1}^{n}[x_i^2-10\cos(2\pi x_i)+10]$	30	[-5.12,5.12]	0
$F_{10}(x)=-20\exp(-0.2\sqrt{\frac{1}{n}\sum_{i=1}^{n} x_i^2}$ $-\exp(\frac{1}{n}\sum_{i=1}^{n}\cos(2\pi x_i))+20+e)$	30	[-32,32]	0
$F_{11}(x)=\frac{1}{400}\sum_{i=1}^{n} x_i^2-\prod_{i=1}^{n}\cos(\frac{x_i}{\sqrt{i}})+1$	30	[-600,600]	0

$F_{12}(x)=\frac{\pi}{n}\{10\sin(\pi y_1) + \sum_{i=1}^{n-1}(y_i-1)^2[1+10\sin^2(\pi y_{i+1})]+(y_n-1)^2\} + \sum_{i=1}^{n}u(x_i,10,100,4), where\ y_i=1+\frac{x_i+1}{4}, u(x_i,a,k,m)=\begin{cases}k(x_i-a)^m & x_i>a\\ 0 & -a<x_i<a\\ k(-x_i-a)^m & x_i<-a\end{cases}$	30	[-50,50]	0
$F_{13}(x)=0.1(\sin^2(3\pi x_1) + \sum_{i=1}^{n}(x_i-1)^2[1+\sin^2(3\pi x_i+1)]) + (x_n-1)^2[1+\sin^2(2\pi x_n)]+\sum_{i=1}^{n}u(x_i,5,100,4)$	30	[-50,50]	0
$F_{14}(x)=(\frac{1}{500}+\sum_{j=1}^{25}\frac{1}{j+\sum_{i=1}^{2}(x_i-a_{ij})^6})^{-1}$	2	[-65.536,65.536]	1
$F_{15}(x)=\sum_{i=1}^{11}[a_i-\frac{x_1(b_i^2+b_ix_2)}{b_i^2+b_ix_3+x_4}]^2$	4	[-5,5]	0.0003075
$F_{16}(x)=4x_1^2-2.1x_1^4+\frac{1}{3}x_1^6+x_1x_2-4x_2^2+x_2^4$	2	[-5,5]	-1.0316285
$F_{17}(x)=(x_2-\frac{5.1}{4\pi^2}x_1^2+\frac{5}{\pi}x_1-6)^2 +10(1-\frac{1}{8\pi})\cos x_1+10$	2	[-5,5]	0.398
$F_{18}(x)=[1+(x_1+x_2+1)^2(19-14x_1 +3x_1^2-14x_2+6x_1x_2+3x_2^2)] \times[30+(2x_1-3x_2)^2\times(18-32x_2 +12x_1^2+48x_2-36x_1x_2+27x_2^2)]$	2	[-2,2]	3
$F_{19}(x)=-\sum_{i=1}^{4}c_i\exp(-\sum_{j=1}^{3}a_{ij}(x_j-p_{ij})^2)$	4	[0,1]	-3.86
$F_{20}(x)=-\sum_{i=1}^{4}c_i\exp(-\sum_{j=1}^{6}a_{ij}(x_j-p_{ij})^2)$	6	[0,1]	-3.32
$F_{21}(x)=-\sum_{i=1}^{5}[(X-a_i)(X-a_i)^T+c_i]^{-1}$	4	[0,10]	-10
$F_{21}(x)=-\sum_{i=1}^{7}[(X-a_i)(X-a_i)^T+c_i]^{-1}$	4	[0,10]	-10
$F_{21}(x)=-\sum_{i=1}^{10}[(X-a_i)(X-a_i)^T+c_i]^{-1}$	4	[0,10]	-10
